# Between Forests and Fields: The Distribution of *Eumorpha* (Lepidoptera, Sphingidae) Moths Across Brazilian Biomes

**DOI:** 10.1007/s13744-026-01362-1

**Published:** 2026-02-05

**Authors:** Tauanny Maria Almeida Lima, José Augusto Teston

**Affiliations:** 1 Programa de Pós-Graduação em Biodiversidade e Biotecnologia (BIONORTE), Universidade Federal do Oeste do Pará, Santarém, Pará Brazil; 2 Laboratório de Estudos de Lepidópteros Neotropicais (LELN), Universidade Federal do Oeste do Pará, Santarém, Pará Brazil

**Keywords:** Amazon, Atlantic Forest, Brazil, Cerrado, Diversity, Insect

## Abstract

The Sphingidae family of moths plays a vital role as pollinators; however, research focusing on certain genera within the family remains limited. This study adopted an integrative approach, drawing on literature, the SiBBr, GBIF, and SpeciesLink databases, and field collections to examine the distribution of *Eumorpha* Hübner, 1807 species across Brazil. A total of thirteen species is known to occur in the country, yet substantial knowledge gaps remain regarding their actual distribution. We compiled 623 occurrence records representing 14 species. Most species and occurrence records were concentrated in the Atlantic Forest and Amazon biomes. The results indicate that research directly focused on *Eumorpha* remains scarce and that, despite advances in the digitization of national collections, the number of related publications has not increased. This study provided important insights into the distribution of *Eumorpha*, showing that some species, such as *Eumorpha vitis* (Linnaeus) and *Eumorpha fasciatus* (Sulzer), have a wide distribution, while others, like *Eumorpha translineatus* (Rothschild), appear more restricted. In addition to contributing to the understanding of *Eumorpha* distribution in Brazil, the study also highlights the relevance of combining multiple data sources to address existing knowledge gaps.

**Supplementary Information:**

The online version contains supplementary material available at 10.1007/s13744-026-01362-1.

## Introduction

Understanding biodiversity is still shaped by significant information gaps, which limit our ability to identify ecological and evolutionary patterns and to guide conservation strategies (Souza et al. 2024). Among these uncertainties are the Wallacean shortfalls, which refer to under-sampled regions and taxonomic groups (Hortal et al. 2015). Beyond being a technical challenge, these gaps directly influence inferences about species distributions, community dynamics, and responses to environmental change (Hortal et al. 2015). In a context where research and conservation resources are limited, recognizing and addressing these shortfalls is essential for directing efforts strategically and ensuring that the knowledge produced is adequate to support management decisions and public policies (Jucker et al. 2018).

The Sphingidae family, commonly known as hawkmoths, comprises a diverse group of moths found throughout all biogeographic regions, except Antarctica. Currently, over 1700 species are recognized, grouped into 213 genera, underscoring the family’s remarkable taxonomic richness (Kitching 2025). The hawkmoths have been the subject of intense research in several areas, notably taxonomy, phylogeny, and biogeography (Kawahara et al. 2009; Haxaire and Mielke 2019; Harshlata et al. 2023; Kitching 2025). This scientific interest stems mainly from their ecological importance, as many species serve as important nocturnal pollinators and are recognized as sensitive bioindicators of environmental change, reflecting wider changes in the composition and dynamics of ecosystems (Kitching and Cadiou 2000; Harshlata et al. 2023).

The genus *Eumorpha* Hübner, 1807 currently comprises 28 recognized species, with a distribution ranging from the Neotropics to the United States, of which 13 are recorded in Brazil (Haxaire and Mielke 2019; Kitching 2025). Although its species are easily recognizable due to their distinctive traits, which include morphologies and important ecological roles, the genus remains relatively understudied. Most available research is restricted to formal taxonomic treatments or species checklists, revealing a notable gap in more comprehensive investigations into its ecology and biogeography (Motta et al. 1998; Kitching and Cadiou 2000; Kawahara et al. 2009; Haxaire 2015; Valente and Teston 2024).

Currently, no *Eumorpha* species are listed as threatened on the IUCN Red List (IUCN 2025); however, moth populations are broadly exposed to multiple anthropogenic pressures, including environmental pollution, climate change, urbanization, industrialization, light pollution, deforestation, and land-use change associated with intensive agriculture and landscape fragmentation (Ferro et al. 2014; Uhl et al. 2021; Vaz et al. 2023; Kumar 2025). In this context, understanding the geographic distribution of species and the processes that shape their spatial occurrence is essential to developing effective conservation strategies (Fox et al. 2014; Meyer et al. 2015; Neff et al. 2025). Furthermore, this knowledge contributes to resolving taxonomic uncertainties and deepening our understanding of the ecological relationships between species and their habitats, particularly in the case of endemic species restricted to specific regions (Gross et al. 2025). This issue is particularly relevant in areas of high biological diversity, such as Brazil, where habitat loss can have serious consequences for the species under investigation (Gross et al. 2025).

In this context, the integration of multiple data sources, including historical records, specialized literature, current georeferenced data, and information from online databases, plays an important role in filling knowledge gaps and supporting future research in ecology, biogeography, and conservation (Marques et al. 2024). The aim of this study was, therefore, to analyze the distribution of *Eumorpha* moth species across Brazilian biomes by integrating multiple data sources.

## Methods

This study was based on five different data sources: scientific literature, field collections, information available in the *Sistema de Informação sobre a Biodiversidade Brasileira* (SiBBr), Global Biodiversity Information Facility (GBIF), and SpeciesLink. For the search of scientific literature, the Scopus, Web of Science, and SciELO databases were used, restricting searches to the title, abstract, and keywords, with data collection conducted up to February 10, 2025. In all databases, the following combination of search terms was used: “*Eumorpha*” OR “Sphingidae” AND “Lepidoptera” AND “Brazil”.

**Fig. 1:**
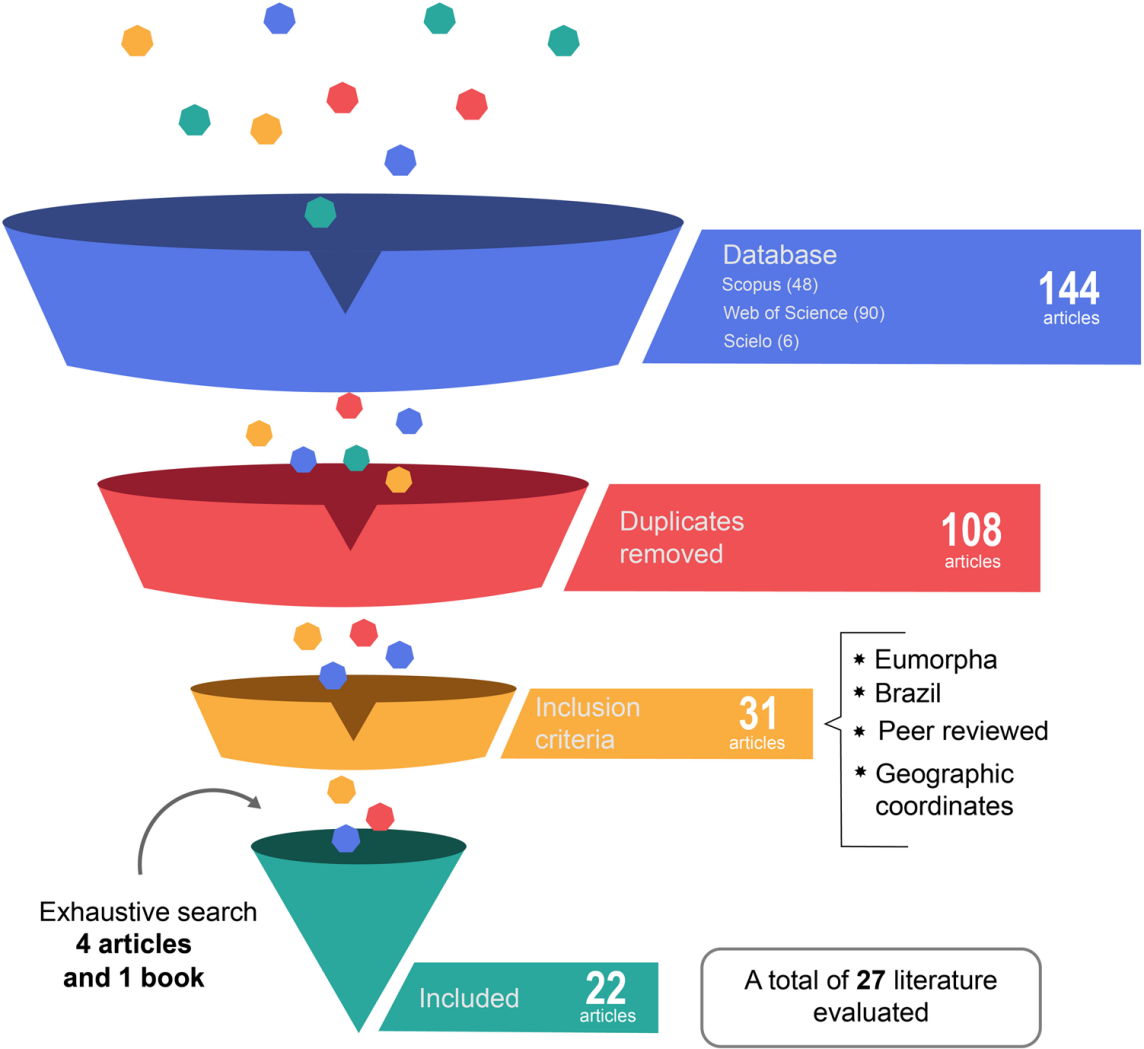
Visual representation of the literature search and filtering according to the inclusion criteria

After obtaining all the studies, a screening was carried out considering first the title and the abstract, then the following criteria were considered: being carried out in Brazil, having a record of occurrence of moths of the *Eumorpha* genus, being peer-reviewed, having geographic coordinates or location information (Fig. 1). For records lacking geographic coordinates but containing information such as municipality, state, or specific locations like protected areas (PAs), centroids were extracted using QGIS software, and the corresponding geographic coordinates were assigned.

In addition to the keyword and Boolean operator–based search for published works, we conducted an exhaustive search that identified four additional articles. This included the manual screening of reference lists, consultation of non-indexed taxonomic literature, and the review of online resources from research institutions. We also opted to include a book, as it provided important information for the execution of this study (Supplementary 1).

### Additional Data

Field collections were carried out from July 2023 to September 2024 in the state of Pará, specifically in the Alter do Chão village, Environmental Protection Area (EPA, 02°30′38″S, 54°56′53″W), the urban zone of Santarém (02°26′19.9″S, 54°44′41.2″W), and a cacao plantation located along the Ramal Chacareiro, a private property in the Uruará municipality (03°41′24″S, 53°46′12.2″W).

In the Alter do Chão EPA, a sheet-type light trap was used, with one monthly collection carried out over five consecutive days. In urban Santarém, material was obtained occasionally. In the cacao plantation, a Pennsylvania-type light trap was employed during two expeditions, conducted in May and September 2023. All collected specimens were deposited to the *Laboratório de Estudos de Lepidópteros Neotropicais* (LELN), located at the *Universidade Federal do Oeste do Pará* (UFOPA), Santarém, where specimens were prepared, mounted, and deposited (Supplementary 1). The specimens were identified using the *habitus* coloration, the specialized literature, and the online catalog Sphingidae Taxonomic Inventory (https://sphingidae.myspecies.info/).

In addition to field collections, data available in the SiBBr, GBIF, and SpeciesLink were also used. The SiBBr dataset was extracted on March 5, 2025, while the GBIF and SpeciesLink datasets were obtained on November 22, 2025. During extraction, the filter “Preserved Specimens” was applied, resulting in an initial total of 855 recorded species. After download, the data underwent a screening and cleaning process, during which all records lacking geographic coordinates and duplicate entries were removed.

### Data Analysis

To represent the flows between data sources, biomes, and species of the *Eumorpha* genus, a Sankey diagram was constructed, highlighting the relationships among these three central axes of analysis. All analyses were performed using Python scripts within the Google Colaboratory environment, employing specific libraries such as Plotly and Pandas. Complementary graphs were also created in Excel to enhance data visualization and support interpretation.

The maps were created using QGIS software, version 3.42.0 (QGIS Development Team 2025), based on shapefiles of Brazilian biomes obtained from the cartographic databases of the *Instituto Brasileiro de Geografia e Estatística* (IBGE). The spatial representations included the distribution of species by biome.

## Results

### Publication History

**Fig. 2:**
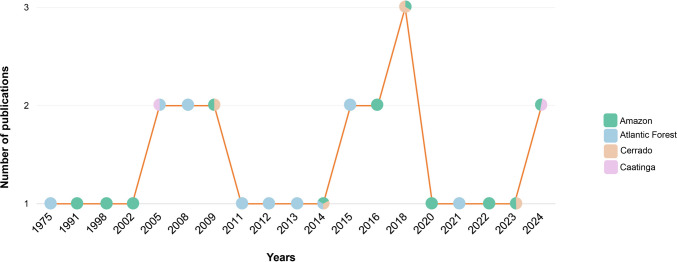
Number of publications reporting records of *Eumorpha* moths over the years

A total of 27 studies were evaluated, corresponding to the period from 1975 to 2024. The year 2018 had the highest number of publications with the focal group, with three publications. Notably, only two of the studies focused specifically on the *Eumorpha* genus, while the remaining works adopted a broader approach centered on the Sphingidae family. When analyzing publications by biome, the Amazon (*n* = 11) and the Atlantic Forest (*n* = 9) were the most represented, followed by the Cerrado (5 studies). The Caatinga had its first record of *Eumorpha* in a study published in 2005, with only one additional study appearing in 2024 (Fig. 2).

When considering the interval between one publication and the next, a notable gap is observed between studies published from 1975 to 2009. From 2011 onwards, however, a more regular though modest pattern of publications has emerged.

### Species Diversity Documented in the Literature

**Fig. 3:**
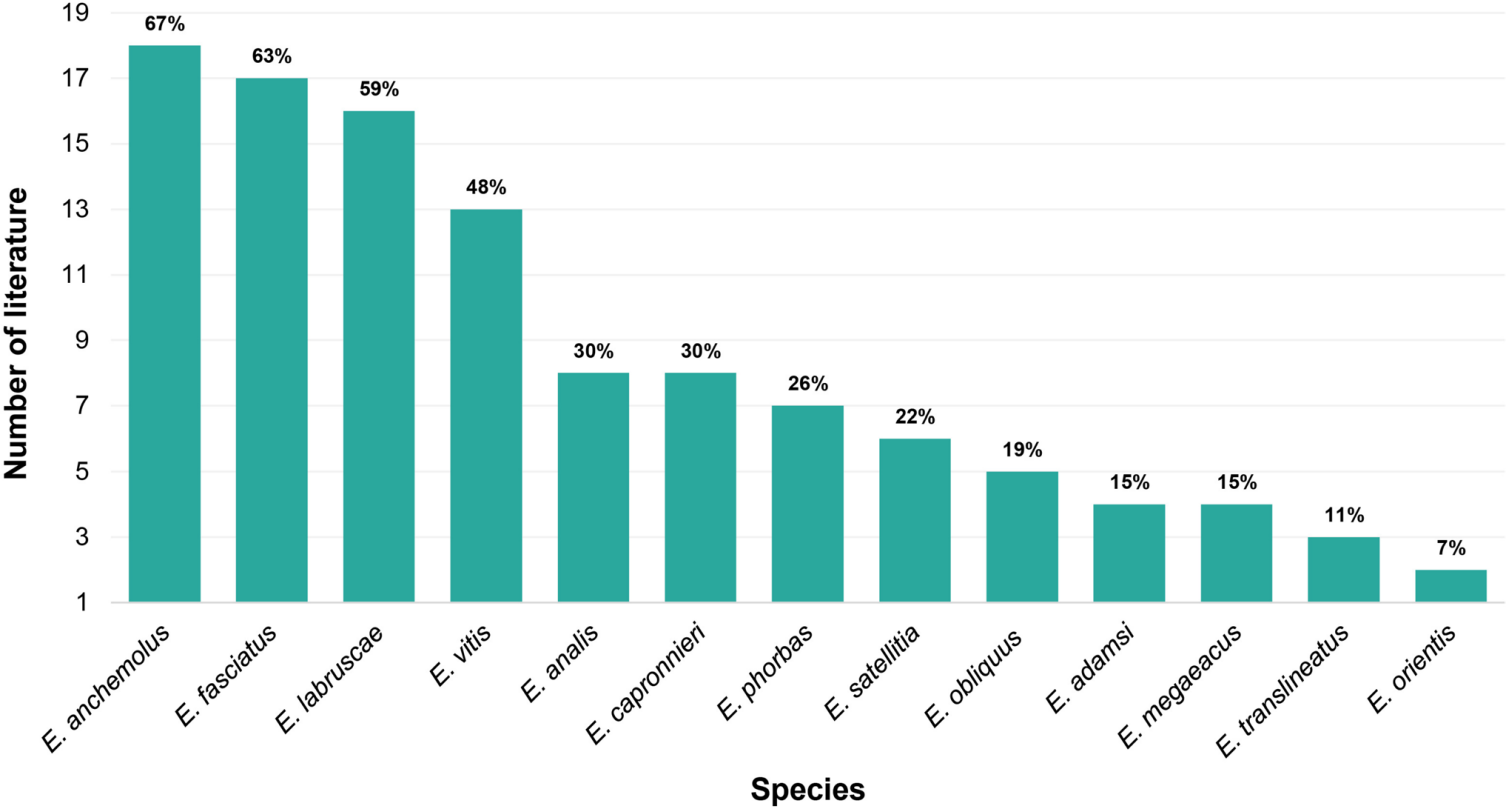
Number of studies recorded for each species of the *Eumorpha* genus

Among the studies evaluated, the most frequently recorded species were *Eumorpha anchemolus* (Cramer) (*n* = 18), followed by *E. fasciatus* (Sulzer) (*n* = 17), *E. labruscae* (Linnaeus) (*n* = 16), and *E. vitis* (Linnaeus) (*n* = 13). The least frequently recorded species in the literature was *E. orientis* (Daniel) (*n* = 2) (Fig. 3).

### Occurrences by Biome

A total of 623 records were obtained from the combined dataset (SpeciesLink: *n* = 302 (48%); literature: *n* = 183 (29%); SiBBr: *n* = 108 (17%); GBIF: *n* = 25 (4%); this study: *n* = 5 (1%)) distributed across 233 unique georeferenced occurrence points. Most occurrence points for the Atlantic Forest (*n* = 63; 53%) and Pampa (*n* = 6; 75%) biomes originated from SpeciesLink, as did the single occurrence point for the Pantanal biome. Regarding the Cerrado biome (*n* = 29, 59%), the majority of occurrence points were sourced from the literature. For the Amazon biome, occurrence points were nearly evenly distributed between the literature (*n* = 20; 36%) and SiBBr (*n* = 19, 34%). In the Caatinga biome, there was no dominant source, with points split between the literature (*n* = 2, 40%), SpeciesLink (*n* = 2, 40%), and SiBBr (*n* = 1, 20%).

**Fig. 4:**
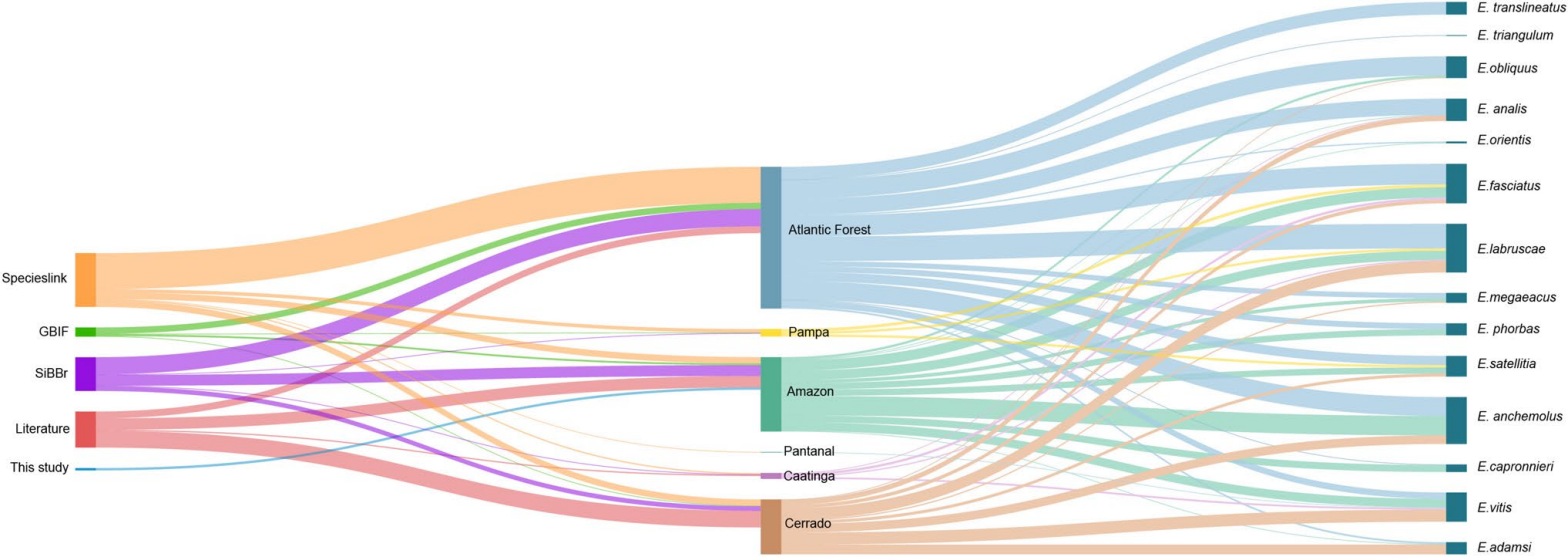
Flows between material source, biome, and species represented in a Sankey diagram

The distribution of species across biomes also showed a clear regional concentration of records in certain regions. For example, *E. adamsi* and *E. vitis* had the majority concentrated in the Cerrado. The species *E. analis*, *E. fasciatus*, *E. labruscae*, *E. megaeacus*, E*. obliquus*, *E. orientis*, *E. phorbas*, and *E. satellitia* were predominant in the Atlantic Forest. *E. capronnieri*, however, stood out for having its occurrences concentrated in the Amazon. Unlike the other species, which had occurrences in more than one biome, *E. translineatus* and *E. triangulum* were restricted to the Atlantic Forest biome (Fig. 4).

Furthermore, there are 14 *Eumorpha* species occurring in Brazil of which all (100%) occurred in the Atlantic Forest, 12 (86%) in the Amazon, nine (64%) in the Cerrado, and four (29%) in the Caatinga. Only three species (14%) were recorded in the Pampa, and a single species was reported for the Pantanal. The species with the highest number of geographic records were *E. anchemolus* (*n* = 86) and *E. labruscae* (*n* = 85), followed by *E. fasciatus* (*n* = 69) and *E. vitis* (*n* = 51), while *E. triangulum* had the fewest occurrence records, with only one georeferenced point. 

**Fig. 5:**
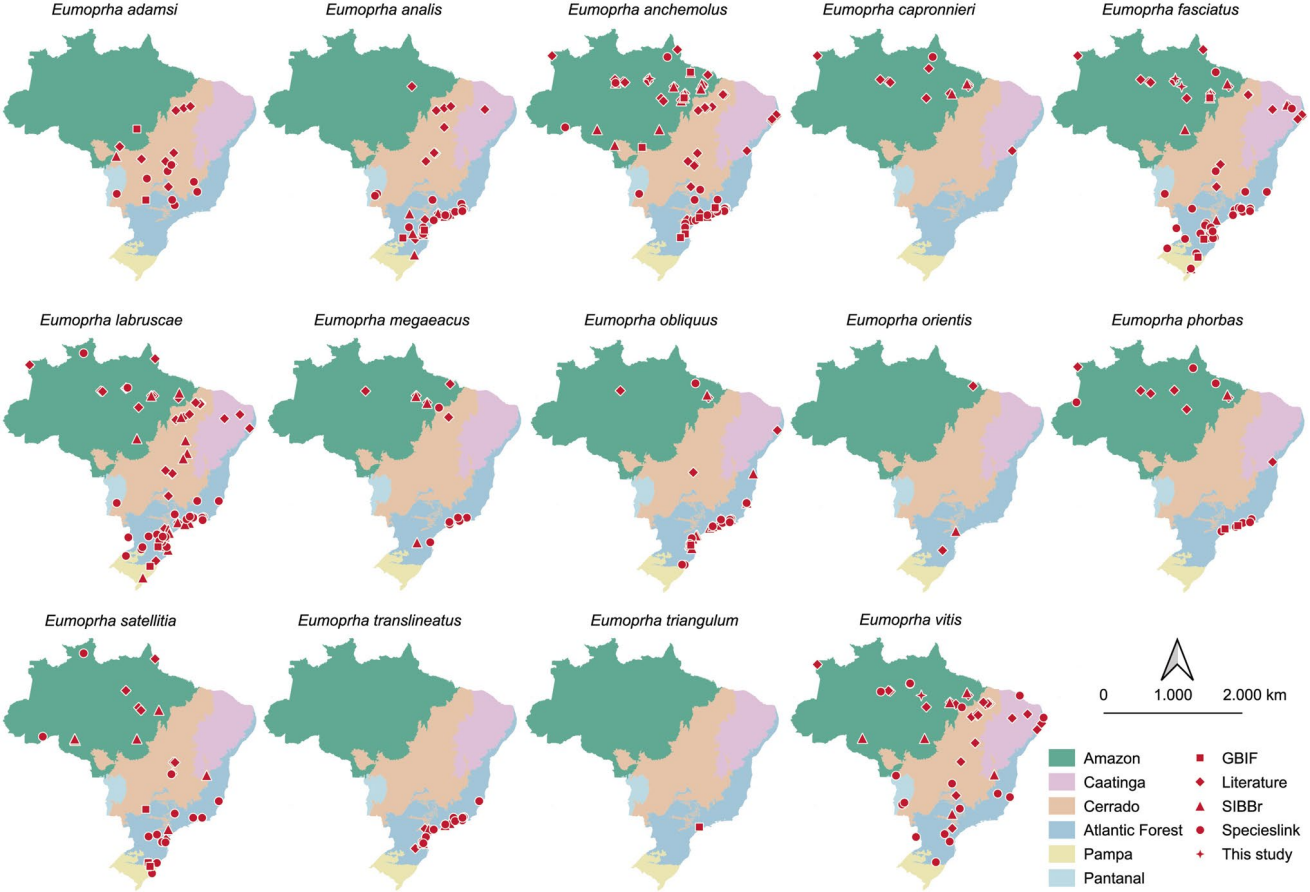
Biogeographic distribution of *Eumorpha* moths in Brazilian biomes

A broad concentration of points can be observed in the southern and southeastern portions of the country, especially in the southern Atlantic Forest, with gaps further north in the biome and near the transition area with the southern Cerrado. In the Cerrado, the records appear more evenly distributed, yet there are still regions with low coverage, particularly in the transition area with the southern portion of the Eastern Amazon. Gaps are also observed in the Pantanal and across much of the Caatinga. In the Amazon, despite a considerable number of occurrence points, there are extensive areas without information, especially in the western portion of the Amazon (Fig. 5).

## Discussion

In this study, we compiled data from various sources and updated the distribution of the *Eumorpha* genus in Brazil. Publications including members of this group remain incipient, with only two studies dedicated exclusively to the *Eumorpha* genus (Ponce et al. 2015; Lima et al. 2023). This scarcity of studies focused on the genus reflects gaps both in taxonomic knowledge and in the ecological and biogeographical understanding of the group.

In many cases, studies with species lists appear as appendices to local or regional faunal inventories (Câmara et al. 2018; Valente and Teston 2024), in which the *Eumorpha* genus, although present, is not the subject of detailed analysis regarding its ecology, distribution, or phylogeny. This often-fragmented approach limits the advancement of systematic and biogeographical knowledge of the group, in addition to restricting the understanding of its ecological roles and its vulnerability to environmental change. Future research should focus on biogeographic, ecological, modeling, and molecular studies to better characterize patterns of diversity, endemism, and distribution that remain largely unexplored within the *Eumorpha* genus.

Another aspect highlighted by the analysis of data obtained from published studies was the uneven chronology and trajectory. The earliest records of *Eumorpha* are concentrated in the Atlantic Forest and Amazon, reflecting the predominance of these biomes in early entomological inventories (see Laroca and Mielke 1975; Motta et al.^1991^, Motta et al.^1998^; Motta & Andreazze 2002). In contrast, historical gaps persist in Caatinga, Cerrado, Pampa, and Pantanal, where sampling has been considerably more limited. This concentration of records in the Atlantic Forest and Amazon biomes also demonstrates the historical predominance of these environments in entomological inventories, often due to their recognized megadiversity and the presence of nearby research institutions (Lazos-Ruíz et al. 2021). Furthermore, it may also be related to the species richness of these biomes (Duarte et al. 2008; Lima et al. 2023).

The first *Eumorpha* moths record in the Caatinga biome appeared in a study in 2005, and another study in 2024 was a study documenting the genus in the region. This gap highlights the historical neglect of biomes considered to have “lower diversity.” Santos et al. (2011) emphasize that the Caatinga has the lowest number of studies and dedicated researchers, especially when compared to the amount of research conducted in humid forests such as the Amazon and Atlantic Forest.

The inclusion of the Cerrado in the literature with genus records occurred even later, in 2009, despite this biome being recognized as a biodiversity hotspot with a high endemism rate (Myers et al. 2000). When it comes to studies on moths, the Cerrado remains greatly neglected compared to other insect groups such as beetles (Coleoptera), bees and wasps (Hymenoptera), and flies (Diptera) (Correa et al. 2025). Despite the recent increase in studies, there is still a need for greater investment, particularly in expanding temporal coverage, increasing sampling effort, and identifying priority areas for understanding the *Eumorpha* fauna in the Caatinga and Cerrado biomes.

These data highlight the overall scarcity of studies on the genus and a historical pattern of unequal research effort among Brazilian biomes, which limits the understanding of the true geographic distribution, ecological requirements, and evolutionary potential of species belonging to the *Eumorpha* genus. However, in recent years, an increase in the number of available records has been observed, which may be associated with expanded digitization of collections and the strengthening of open data platforms (Hedrick et al. 2020). Nevertheless, the analytical use of these data remains limited as standardized formats for online available data are still lacking (Feng et al. 2022). The high frequency of records for certain species in publications, such as *E. anchemolus*, *E. fasciatus*, *E. labruscae*, and *E. vitis*, may be related to multiple ecological and methodological factors. These species generally exhibit attributes that increase detectability in faunal surveys, as taxa with wide geographic distribution and high abundance tend to be more easily recorded (Gaston and Lawton 1988).

Evidence suggests that forest corridors intermittently connected the Amazon and Atlantic Forest in the past (Por 1992), facilitating species exchange, especially among highly mobile groups like hawkmoths, which serve as key pollinators and bioindicators in tropical ecosystems (Cerdal et al. 2002). Despite their ecological importance, we showed that the distribution of many hawkmoth species remains poorly understood.

Species geographical distribution of the *Eumorpha* genus in Brazil exhibits distinct patterns of occurrence across different biomes, including cases of geographically disjunct distributions, and also with consistent indications that some species may represent complexes due to their cryptic nature, as suggested by Eitschberger (2011) and Lima et al. (2023). When these patterns are analyzed alongside historical occurrence data, they reinforce the importance of integrative approaches for delimiting taxonomic and biogeographic groups.

The species *E. capronieri*, *E. phorbas*, *E. megaeacus*, and *E. orientis* exhibited disjunct distributions. Patterns of disjunct distributions may raise the possibility of population differentiation along environmental gradients or even following historical fragmentation events (Sgarlata et al. 2025). In Lepidoptera, isolated populations may, in some cases, correspond to divergent lineages or even subspecies, especially in groups with strong associations to specific habitats (Sourakov and Zakharov 2011; Jiang et al. 2016; Vargas-Ortiz et al. 2018). However, for the species treated in this study, no formally described subspecies correspond to these isolated population nuclei (Kitching 2025). Moreover, phylogenetic or phylogeographic studies available for Sphingidae remain limited and, when present, lack sufficiently broad geographic sampling to assess whether these populations represent distinct evolutionary units (Kawahara et al. 2009).

Species such as *E. labruscae*, *E. fasciatus*, and *E. vitis* were found to be widely distributed, with occurrence records across multiple biomes, including urban areas (Haxaire and Mielke 2019). Their ubiquity appears to be related to high ecological plasticity and the ability to adapt to anthropized environments. *E. adamsi*, although recorded in the Amazon and Atlantic Forest, showed its highest concentration in the Cerrado, likely benefiting from the environmental heterogeneity of the biome, which allows the creation of diverse microhabitats and supports complex ecological interactions (Ratter et al. 2010; Pacheco and Vasconcelos 2012; De Brito Freire et al. 2024; Cardoso et al. 2025).

It was also possible to observe the restricted distribution of some species, such as *E. tanslineatus*, limited to the southern Atlantic Forest (Haxaire and Mielke 2019). This geographic restriction may be related to greater ecological specialization of the species or may reflect under-sampling in other regions. Accurate delineation of these occurrence areas will depend on broader and more systematic surveys, especially in transition zones between biomes. *Eumorpha triangulum* also had a record for the Atlantic Forest; however, since this is the first time the species has been reported for Brazil and the record comes from a database entry, caution is recommended regarding its actual occurrence in the country.

Several taxa likely represent as species complexes, including *E. analis*, *E. anchemolus*, *E. obliquus*, and *E. orientis*, based on genitalia morphological variations (Eitschberger 2011; Lima et al. 2023). These complexes highlight the cryptic diversity within the genus, emphasizing the need for a comprehensive taxonomic revision to clarify species boundaries and more precisely define their geographic distributions.

Despite the data compiled here, this study still presents important limitations. The field collections conducted over a relatively short interval may not fully capture temporal variations in species activity, detectability, or local abundance, especially in groups with seasonal dynamics. Likewise, occurrence records extracted from the literature and online repositories often reflect heterogeneous sampling efforts, taxonomic uncertainties, and spatial gaps—factors that can influence the observed distribution patterns. Although these datasets are complementary, the integrated approach may still underrepresent poorly sampled regions or rare species.

## Conclusion

The distribution of the *Eumorpha* genus in Brazil reflects both the group’s complexity and the knowledge gaps accumulated over time. Although recent data and digital platforms have expanded access to occurrence records, scientific production remains sporadic and geographically uneven, reflecting historical sampling patterns concentrated mainly in the Amazon and Atlantic Forest biomes. Biomes such as the Caatinga, Cerrado, and especially the Pampa and Pantanal remain under-sampled, limiting a more accurate understanding of species diversity and distribution.

In this context, the occurrence of species with apparently disjunct distributions, such as *E. phorbas*, *E. orientis*, *E. megaeacus*, and *E. capronnieri*, should be interpreted with caution, as these patterns may be influenced by limited sampling, taxonomic uncertainty, and the absence of formally recognized subspecies, in addition to potential misidentifications among morphologically similar taxa. Another point requiring caution concerns occurrence records of *E. triangulum*, which may further reflect identification uncertainties and incomplete sampling coverage.

Widespread species such as *E. labruscae*, *E. fasciatus*, and *E. vitis*, including records in urban areas, demonstrate high ecological plasticity. However, their presence across different biomes and observed morphological variations reinforce the need for integrative approaches combining morphology, genetics, and ecological data for more accurate taxonomic revision. Thus, this study contributes to the understanding of the diversity and distribution of *Eumorpha* species in Brazil and highlights the importance of continuous and multidisciplinary research to elucidate the true diversity of this group within the national context, mainly in areas with little or no study yet carried out.

## Supplementary Information

Below is the link to the electronic supplementary material.

ESM1 (XLSX 61.3 KB)

## Data Availability

All data are provided in the supplementary materials.
